# Effect of the Polar Head Type on the Surface Adsorption and Tribofilm Formation of Organic Friction Modifiers in Water-Based Lubricants

**DOI:** 10.1021/acs.langmuir.3c03729

**Published:** 2024-04-04

**Authors:** Tanaelle Marmorat, Wahyu Wijanarko, Nuria Espallargas, Hamid Khanmohammadi

**Affiliations:** Norwegian Tribology Center, Department of Mechanical and Industrial Engineering, Norwegian University of Science and Technology (NTNU), Trondheim, 7491, Norway

## Abstract

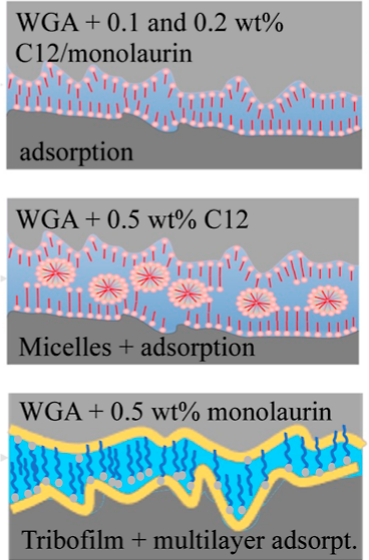

Carboxylic acids make up a well-known group of organic friction modifiers (OFMs). OFMs can present different types of polar heads that can eventually lead to different surface adsorption properties and tribological responses. Therefore, the goal of this work is to study the effects of the polar head type on the frictional and wear performances of carboxylic acids in a water-based lubricant. Lauric acid (C12) was chosen as the reference OFM, and methyl laurate and monolaurin were chosen for the comparison. Sliding friction tests were performed on stainless steel against alumina balls under boundary lubricating conditions. The effect of the adsorbed layers and the tribofilm formation was studied by varying the initial maximum hertzian contact pressure, i.e., tests were performed at 1.97 and 0.66 GPa. At the lowest contact pressure, not enough load is applied to obtain enough plastic deformation on the asperity contacts. In this case, a combination of asperity contacts and a thick fluid film formation results in a lack of tribofilm formation, whereas at the highest contact pressure, tribofilms are formed in the asperity contact through tribochemical reactions. Methyl laurate showed no adsorption on the surface, and it was not tested further. C12 and monolaurin showed good adsorption, but the adsorbed layers had different viscoelastic properties. Micro and macrotribological tests showed good frictional behavior for C12 at 0.5 wt % concentration due to the good viscoelastic properties of its adsorbed layer. The adsorbed layer of monolaurin did not show good friction-reducing ability during the micro tribological tests due to its poorer viscoelastic properties. However, the macro tribological tests revealed that monolaurin forms a robust tribofilm protecting the surface from wear and efficiently reducing friction at a concentration of 0.5 wt % resulting in the lowest wear and friction values as observed in this study.

## Introduction

About 23% of the world’s total energy consumption originates from tribological contacts, of which 20% is used to overcome friction and 3% originates from wear and wear-related failures.^1−3^ Therefore, if we want to improve these figures for a true green transition, better lubrication is needed in moving parts to reduce friction and wear losses. Lubricants consist of about 70–99% base oil and 1–30% chemical additives, depending on the application and use of the lubricant. Base oils are commonly based on hydrocarbons and have two main sources: biological (animal or vegetable sources) and nonbiological (mineral or synthetic oils produced from crude oil). This results in a broad variety of base oils with polar or nonpolar nature. Additives are hydrocarbon or inorganic chemical compounds added to the base lubricant either to enhance an existing property (pour point, oxidation resistance, and viscosity) or to add a new property (anticorrosion, friction control, and antiwear performance). In addition to the current concern about energy losses, there is also nowadays an increased concern about the toxicity of chemical substances used in lubricants, in both the base oils and the additives. Indeed, almost 75% of the base oils (groups 1, 2, and 3) are toxic substances, and many of the most common additives used by lubricant formulators have shown acute toxicity effects and bioaccumulation problems.^4,5^

To overcome the above-mentioned challenges, environmental legislations and society concerns are pushing different industries toward switching from conventional mineral oil-based lubricants to environmentally acceptable lubricants (EALs), although the shift is not happening as fast as the society needs.^4−8^ Among the different alternatives to conventional lubricants, water-based lubricants (WBLs) are promising candidates for EALs. They have some desirable properties, such as good cooling ability, low toxicity, biodegradability, and fire resistance. Indeed, they are already used as fluids for metal cutting^9,10^ and hydraulic systems,^11−13^ and water alone is used as lubricant in polymer and composite bearings in propeller shafts of ships.^14−16^ However, in more demanding triboapplications, water-based lubricants have several drawbacks such as low viscosity, poor corrosion resistance, and incompatibility with lubricant additive packages and seals, leading to poor lubricating performance compared to mineral and synthetic oils. However, low viscosity will be an advantage in the future since more and more lubricated systems are in need of improving their frictional efficiency. Thus, it is crucial to increase the research efforts toward improved WBL formulations. Current lubricant additive packages have been developed and optimized through decades to perform best in mineral oils and might not be compatible or perform best in WBLs. Therefore, new additives should be formulated, and current additives should be further studied to address the specific needs of water-based lubricants.

Friction modifiers (FMs) are at the highest priority among the types of additives that need to be optimized for WBLs because of the competition for surface adsorption sites with other polar substances in the lubricant formulation. There are three main groups of FMs, organic friction modifiers (OFMs), organomolybdenum friction modifiers, and polymer friction modifiers. Among these, OFMs are an example of low toxicity friction modifier additives; more specifically, most anionic and nonionic OFMs are nontoxic. OFMs are typically surface active substances (surfactants) with an amphiphilic structure, i.e., they have a hydrophilic (polar) head and a hydrophobic (nonpolar) tail. Carboxylic acids are a well-known type of surfactant that represent an important group of OFMs for reducing friction under boundary lubricating conditions. The friction reduction mechanism of carboxylic acids is related to their amphiphilic nature, with the polar head adsorbing on the metal surfaces of the tribopair and the nonpolar tail extending out to the bulk of the base lubricant.^17^ These additives physically adsorb on metal surfaces forming mono or multilayer structures preventing true contact between the sliding surfaces through steric hindrance mechanisms.^18,19^ However, in water media, they present several challenges such as their reaction with bivalent metal ions in solution that result from external, or in situ generated contamination, leading to the increase in friction due to the desorption of FMs from the metal surface.^18^ Other challenges are the competition for surface adsorption sites when other polar substances (e.g., amines, water, and thickener) are present in the lubricant formula and their lower solubility in aqueous media with increasing hydrocarbon chain length, among others. Interestingly, carboxylic acids as friction modifier additives in water-based lubricants have not been widely studied despite being a good environmentally friendly alternative and the many advantages of those in water media.^17,20^ Therefore, further research is needed to find the right additives to successfully formulate WBLs.

The antiwear performance of lubricants is also a very important function to control in a tribosystem to improve energy losses. This function is typically achieved by other groups of chemicals than the ones of FMs through the formation of a protective tribofilm that ultimately controls friction and wear at the tribosurface. This mechanism is activated by in situ mechanochemical reactions between species from the lubricant and the metallic elements of the tribosurface.^21,22^ Not surprisingly, the ability of OFMs to form tribofilms has attracted little attention,^23,24^ and most studies focus on their adsorption and frictional performance.^17,25−27^ However, in former works performed in our group, the ability of OFMs to form protective tribofilms in WBLs has been observed.^21,22,24,28,29^ If FMs can lead to both friction and wear reduction, they can eventually have a double function (multifunction) in the lubricant, leading to simpler and greener formulations.

In this work, we study dodecyl alkyl chain surfactants with different polar heads. The choice of the polar head is made based on the expectation to obtain different surface adsorption properties and therefore different frictional and wear performances. Three different surfactants have been chosen for testing in a WBL consisting of water and glycol: (1) lauric acid (C_12_H_24_O_2_) as a reference of a well-known anionic organic friction modifier used in hydraulic fluids; (2) methyl laurate (C_13_H_26_O_2_), a fatty acid methyl ester nonionic surfactant that replaces the H atom in the OH group of the lauric acid by a methyl group; and (3) monolaurin (C_15_H_30_O_4_), a monoester nonionic surfactant formed from glycerol and lauric acid. Lauric acid is inexpensive and nontoxic, and it is therefore safe to handle and finds many uses in the cosmetic industry. Lauric acid has some potential antimicrobial properties; however, it is difficult to dissolve in water at high concentrations and requires an alkali pH for that purpose. Methyl laurate is a less commonly used surfactant for OFMs and is mostly found in the production of biodiesel. Monolaurin is a very common surfactant used in the cosmetic, food, and medical industries due to its strong antifungal, antiviral, and antibacterial properties. The effect of the concentration of two of the three surfactants (lauric acid and monolaurin) has also been studied in this work. The frictional response of the lubricants was tested both at the microscale (applying very low loads to only slide on the OFM adsorbed layer) and at the macroscale (high contact pressure in boundary lubricating conditions). The comparison of the micro- and macro responses reveals the role of the adsorbed layer on both the friction and the tribofilm formation. The wear performance was only studied at the macroscale tests since no wear was observed in the microscale tests.

## Experimental Procedure

### Materials

AISI 316L grade austenitic stainless steel was chosen as the tribomaterial since this is an alloy we have used in our research group in the last years due to its good compatibility with WBLs and its extended use in maritime components.^17,21,22,28^ The samples were cut from a rod with a diameter of 30 mm and were ground using SiC papers of up to 4000 grit followed by polishing in a suspension containing 6 μm diamond particles to obtain a surface finish of *R*_a_ = 0.090 ± 0.004 μm. The polished disks were cleaned ultrasonically in ethanol for 5 min followed by rinsing with fresh ethanol and drying with pressurized air.

The water-based lubricant (WG) was prepared with a mixture of 50–50 wt % distilled water and glycol with a viscosity of 75 mPas at 25 °C. The final viscosity of the water–glycol mixtures at 25 °C was 9.86 mPa s. The glycol alone has a pour point of −39 °C and a flash point of 124 °C. For the preliminary tests, 0.1 wt % of the additives (Table 1) were added to the base lubricant, and the mixtures were prepared by magnetic stirring at 40 °C for 2 h. The solubility of the additives was checked with the naked eye after time periods of 1, 5, 24, and 48 h. The effect of additive concentration on the frictional and wear performance of the lubricant was studied with C12 and monolaurin at concentrations of 0.1, 0.2, and 0.5 wt %. Alkaline pHs are needed for dissolving higher amounts (>0.1 wt %) of OFMs in aqueous solutions.^17,28^ Thus, 1 wt % of *N*,*N*-dimethylethanolamine was added to the base lubricant for this part of the work. The lubricant mixtures containing amine were labeled as WGA and have also been used to study the surface adsorption competition between OFMs and amine, which is also a polar compound that functions as a corrosion inhibitor. Table 2 shows the pH and viscosity values measured in all lubricants formulated for this work. Viscosity measurements were performed using an Anton Paar SVM 3001 viscosimeter.

**Table 1:**
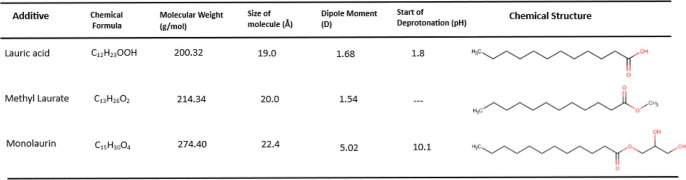
Chemical Formula, Chemical Structure, and Molecular Weight of the Additives Used in This Study^a^

a The onset pH for the deprotonation and the dipole moment were calculated by using Marvin software.

**Table 2:** pH and Viscosity Measurements of all Lubricants Formulated for This Work

	WG	WG0.1C12	WG0.1 monolaurin	WGA	WGA0.1C12	WGA0.2 C12	WGA0.5 C12	WGA0.1 monolaurin	WGA0.2 monolaurin	WGA0.5 monolaurin
PH	7.8	5.8	7.7	10.9	9.9	9.6	9.4	10.8	10.8	10.7
viscosity (mPa s)	9.86	9.91	9.96	10.11	10.16	10.19	10.28	10.16	10.18	10.23

### Testing and Characterization Methods

The surface adsorption studies were performed using a *Quartz crystal microbalance with dissipation mode* (QCM-D) supplied by *Biolin Scientific*. Stainless steel-coated AT-cut 5 MHz sensors from *QSense* (*Biolin Scientific*) with a surface roughness less than 1 nm were used. The flow rate was maintained at 0.05 mL/min during the experiments using a peristaltic pump. The resonance frequency and dissipation shift for the fundamental frequency and third, fifth, and seventh overtones were recorded. MATLAB was used for developing a code to model the viscoelastic properties of the adsorbed layers using the Voigt model.^30−32^ More details about performing the QCM-D tests and viscoelastic modeling of the adsorbed layers have been discussed elsewhere.^21^

The tribotests were performed using a *Rtec MFT-5000* tribometer. For the macrotests, a 6 mm diameter alumina ball was used against the stainless-steel disks. The applied normal load was 20 N, resulting in an initial maximum Hertzian contact pressure of 1.97 GPa. The disk’s rotational speed and the track diameter were set as 0.016 m/s and 10 mm, respectively, and the duration of the macrotests was 30 min. In the case of the microtests, the nanomodule of the *Rtec MFT-5000* tribometer was used. A 1/16″ alumina ball attached to a stiff cantilever was utilized against the polished stainless steel surface with a reciprocating motion. A normal load of 50 mN was applied to the ceramic ball through the metallic cantilever, resulting in an initial maximum Hertzian contact pressure of 0.66 GPa. The stroke length, frequency, and test duration were set to 10 mm, 1 Hz, and 6 min, respectively. The friction data of the microtests were averaged by the *Rtec Viewer* software over 50% of the cycle around the midpoint at the maximum linear speed. In both cases, the tests are performed under boundary lubricating conditions.

*Alicona Infinite Focus 3D Microscope* was used to provide 3D topographic images from 4 different points of the wear tracks, and the cross-sectional images were analyzed using *MountainsMap* software to quantify the wear volume.

A *FEI Quanta 650* scanning electron microscope (SEM) was utilized to study the top surface of the wear tracks, and *Helios G5* plasma focused ion beam (PFIB) was utilized to acquire cross-section images from the subsurface area of the wear tracks. The PFIB-SEM was also used to prepare thin wear track lamellae with thicknesses of about 60 nm. These lamellae were studied in the same PFIB instrument without breaking the vacuum by using a retractable scanning–transmission electron microscopy (STEM) detector. The chemical composition of the lamellae was studied by means of an energy dispersive spectrometry (EDS) detector.

## Results

### Adsorption of the Additives on Stainless Steel in the Absence of Amine

**Figure 1:**
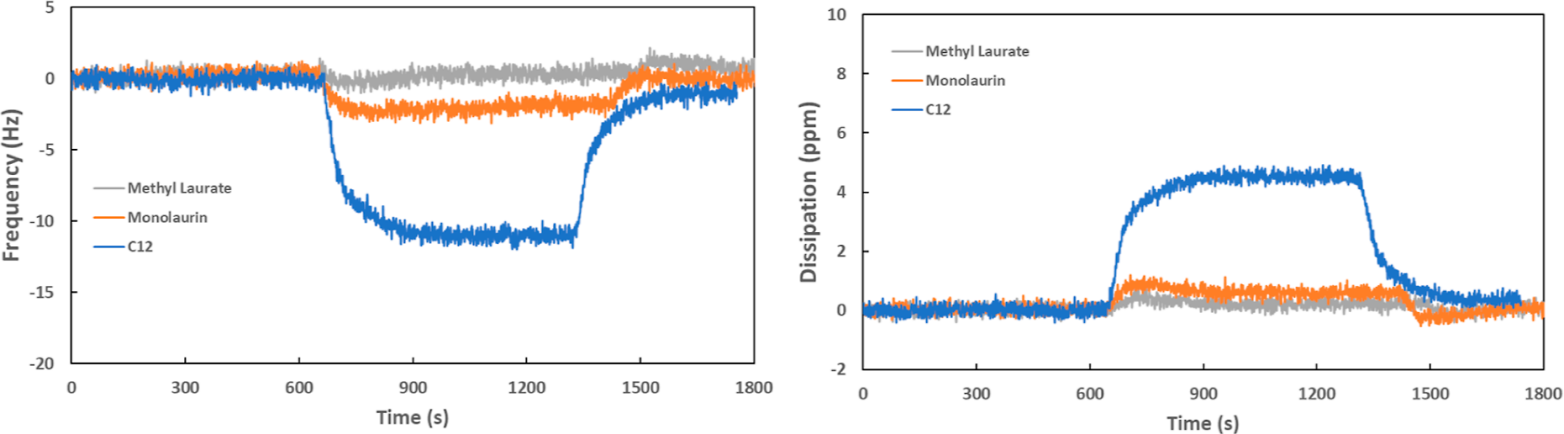
Changes in the frequency and dissipation of the third overtone during the test sequence of WG → WG + Add → WG.

In the adsorption studies of C12, monolaurin, and methyl laurate in the base lubricant without amine (WG), the QCM tests were started by circulating the base fluid (WG) for about 600 s, then combining it with the additivated lubricant (WG + 0.1 wt % additive), and finally rinsing the sample with the base lubricant (WG). Figure 1 shows the changes in frequency and dissipation of the third overtone (f3 and D3) during the mentioned sequence. C12 showed the highest frequency drop (about 12 Hz in the third overtone) followed by monolaurin (about 3 Hz); however, methyl laurate did not show any change in the resonance frequency and dissipation. By checking the onset pH for the deprotonation for these three surfactants (Table 1), it is found that C12 deprotonates easily at neutral pH, monolaurin deprotonates at pH > 10, and methyl laurate remains neutral at all pH ranges (because it cannot deprotonate). This can clearly explain the adsorption behavior in Figure 1 since the pH of the WG mixtures is around 7 (Table 2). The small frequency drop for monolaurin can be due to its very high dipole moment, the presence of oxygen atoms in its structure, and the lack of deprotonation at neutral pH (Table 1). Since C12 is already deprotonated at a neutral pH, the frequency drop is the largest for all OFMs studied in this work. However, methyl laurate does not deprotonate at all, and therefore, it does not effectively interact with the metal surface showing no changes in the frequency drop.

### Friction and Wear in the Absence of Amine

**Figure 2:**
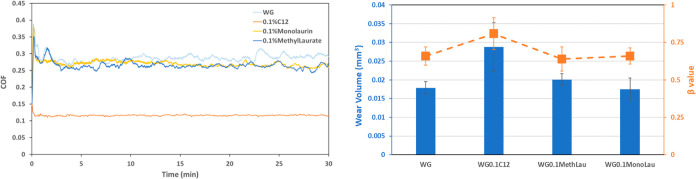
Friction evolution (left), wear volume, and β value (right) for the tests in WG base fluid.

Figure 2 shows the evolution of friction, wear volume, and β value (the degree of material loss) during the macrotests performed in the WG base fluid and WG containing 0.1 wt % of additives. The degree of the material loss is defined as the ratio between the area loss and the groove area, and its determination has been published somewhere else.^29,33^

The friction evolution plots show a coefficient of friction around 0.3 for the base WG lubricant and almost the same value for the base lubricant (WG) with methyl laurate and monolaurin. The lubricant containing C12 shows a very stable and low coefficient of friction around 0.12 with a very short running in period. C12 in WG shows a satisfactory functionality as a friction modifier, whereas methyl laurate and monolaurin do not show any friction modification ability. The poorer friction modification ability for monolaurin and methyl laurate can be explained by the QCM-D results in Figure 1, where poorer surface adsorption was found for these two surfactants. Therefore, a minimum adsorption is needed to act as a friction modifier under boundary lubricating conditions.

On the other hand, the wear volume and β values show the highest material loss for C12 and almost the same wear behavior for the three other lubricants tested. Based on these results, further research will follow only with C12 and monolaurin because they were the only additives adsorbing on the surface of stainless steel.

### Friction and Wear in the Presence of Amine

The effect of the additives’ concentration was tested by adding 1 wt % *N*,*N*-dimethylethanolamine to the base mixture (WGA) to increase their solubility in aqueous solution. This is indeed a typical strategy in formulating commercial WBLs such as hydraulic fluids for increasing the solubility of some additives in addition to keeping a high pH level to avoid corrosion of iron-based components. Three different concentrations (0.1, 0.2, and 0.5 wt %) of C12 and monolaurin were added to the WGA. The reason for choosing the mentioned concentrations is that the critical micelle concentration (CMC) of C12 in water is higher than 0.2 wt %.^17^

**Figure 3:**
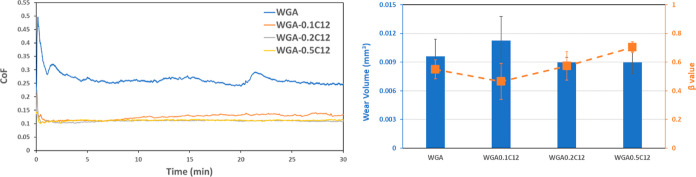
Friction evolution (left), wear volume, and β value (right) for the tests in WGA with different concentrations of C12.

Figure 3 illustrates the friction evolution, wear volume, and β values for the WGA base fluid alone and with different concentrations of C12. Adding 1 wt % of the amine did not affect friction compared to WG, but WGA0.1C12 shows slightly higher friction toward the end of the sliding period than the same lubricant without amine (WG0.1C12). This can be due to the competitive adsorption between amine and carboxylic acid on the surface. As the C12 concentration increases, friction decreases and stabilizes to friction values around 0.11. Comparing the wear volumes reported in Figures 2 and 3, it is found that adding 1 wt % amine resulted in a drastic decrease in wear for all cases (from 0.0179 mm^3^ in WG to 0.0096 mm^3^ in WGA). Increasing the concentration of C12 decreases wear, with the β value showing the reverse trend.

**Figure 4:**
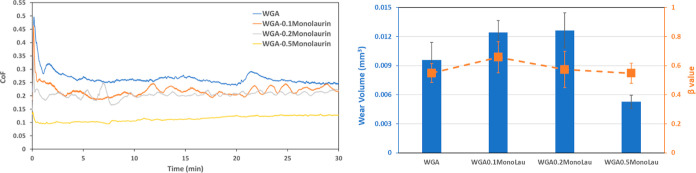
Friction evolution (left), wear volume, and β value (right) for the tests in WGA with different concentrations of monolaurin.

Figure 4 shows the friction evolution, wear volume, and β values for the WGA alone and with different concentrations of monolaurin. Adding 0.1 and 0.2 wt % monolaurin did not have a significant effect in reducing friction, and it indeed increased the wear with respect to WGA. The effect was very similar to what was found in the absence of amine (Figure 2). However, the friction significantly decreases at 0.5 wt % monolaurin to similar values to C12 (Figure 3). In addition, 0.5 wt % monolaurin results in the lowest wear volume of all tests performed in this work.

### Adsorption of the Additives on Stainless Steel in the Presence of Amine

**Figure 5:**
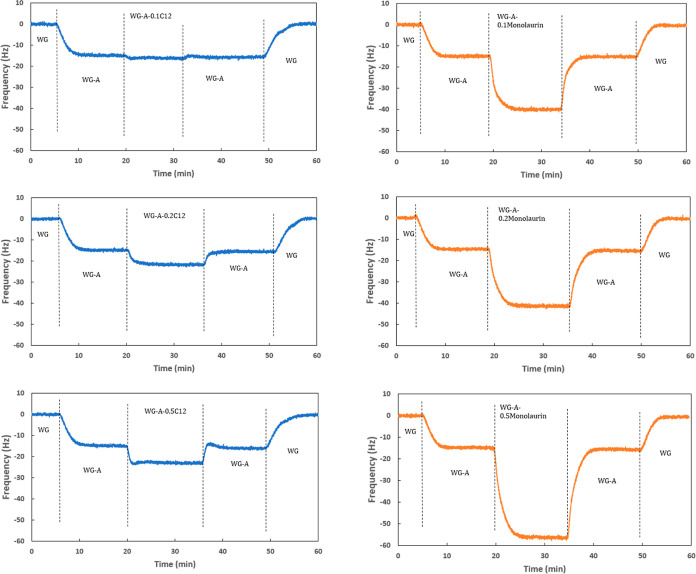
Frequency evolution during QCM-D measurements of different concentrations of C12 (left) and monolaurin (right).

The surface adsorption competition between the amine and the OFMs was studied by using QCM-D. Figure 5 shows the frequency evolution of the lubricants containing different concentrations of C12 and monolaurin in WGA.

**Figure 6:**
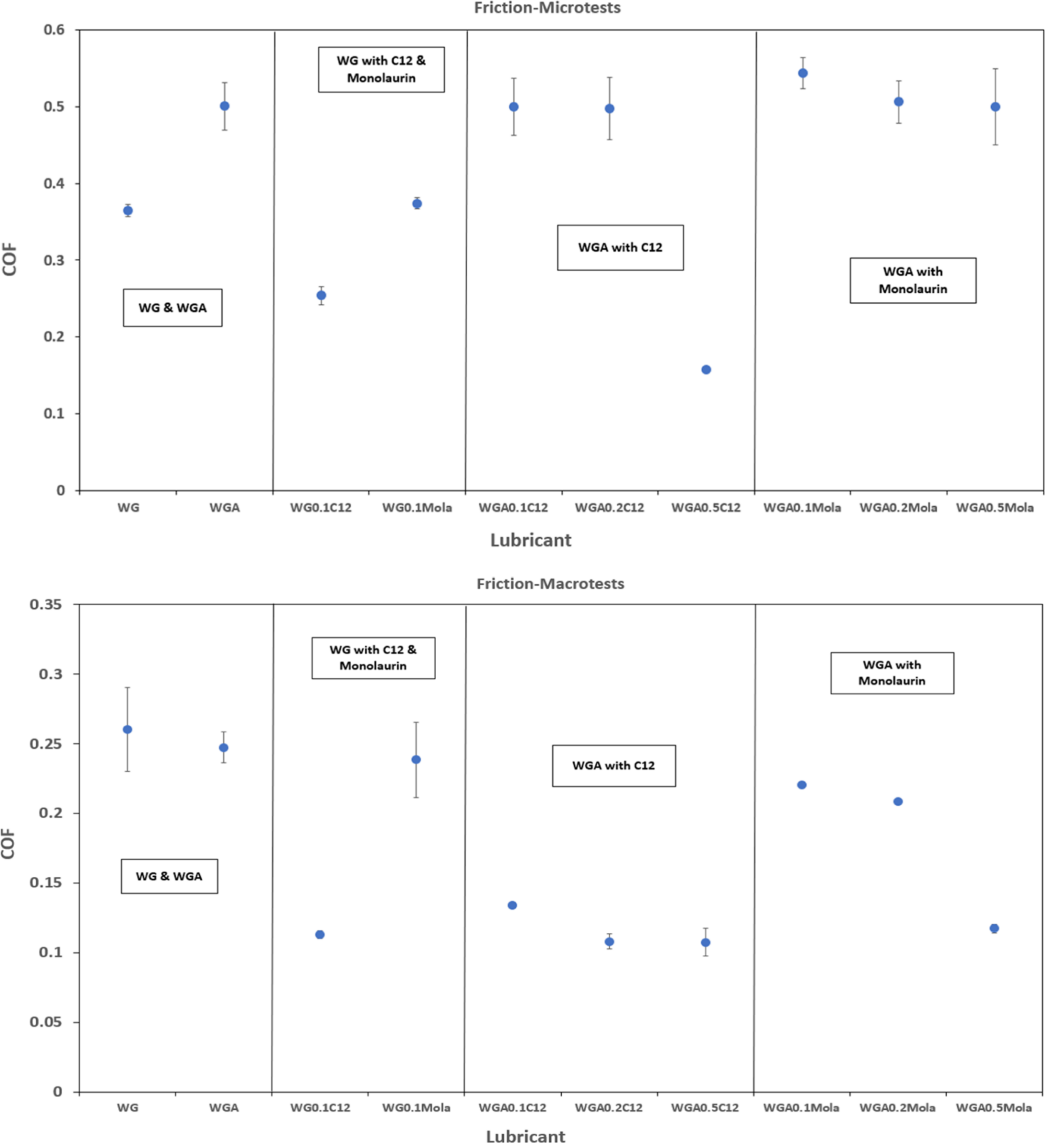
Average coefficient of friction during micro- and macrotribotests.

As can be seen, switching from WG to WGA resulted in a frequency drop of about 15 Hz. This is because of the adsorption of the amine molecules to the surface of the stainless-steel coated sensor. In the case of C12, the resonance frequency slightly decreases by switching from WGA to WGA with 0.1 wt % C12. This can be due to the competitive adsorption between the amine and C12 on the surface, resulting in the replacement of some amine molecules with C12. The total mass change on the surface is not significant, probably because of the arrangement of the molecules on the surface or even because of poor surface coverage. In the case of 0.2 wt %C12, there is an obvious reduction in the frequency, and for 0.5 wt % C12, a sharp reduction of about 10 Hz is found. It should be noted that the CMC of C12 in water is slightly above 0.2 wt % in the WGA mixture;^28,34^ therefore, at 0.5 wt %, the CMC value is greatly exceeded, which is indeed reflected in the different QCM responses in Figure 6. Indeed, the presence of micelles can be seen by the changes in viscosity of the lubricants (Table 2). Surface adsorption competition might still exist between the micelles and the amine, but since the size of the micelles is larger than the C12 brushes alone, a larger reduction in the frequency is found by QCM. In the case of monolaurin, there is an obvious frequency drop of about 25 Hz already at a concentration of 0.1 wt % monolaurin. Therefore, no surface adsorption competition with the amine takes place. This drop does not change at 0.2 wt % monolaurin, which can be the reason for the similar frictional response of 0.1 and 0.2 wt % monolaurin in WGA (Figure 4). On the other hand, when the concentration of monolaurin increases to 0.5 wt %, the resonance frequency drops to about 40 Hz (Figure 5), which can explain its better frictional response. Indeed, monolaurin molecules are fully deprotonated and carry a stronger negative charge (very high dipole moment) than C12 in WGA (Table 2) resulting in higher adsorption on the positively charged metallic surface. The better adsorption of monolaurin in WGA can therefore be attributed to the deprotonation and the high dipole moment of the molecule at alkaline pH.

By considering both adsorption and tribological results, it can be concluded that despite the higher adsorbed monolaurin mass, C12 shows better frictional functionality. However, the higher adsorption of monolaurin at any concentration contributes to a better wear performance. These phenomena will be discussed in more detail in the upcoming sections with the help of the microtests.

## Discussion

### Friction at Micro and Macrocontact Levels

As already mentioned in the introduction, adsorbed layers and tribofilm formation are the two main phenomena affecting friction and wear. In order to study this in detail, the triboloical response (friction and wear) obtained in the microtests (boundary lubricating regime at low contact pressure) and macrotests (boundary lubricating regime at a high contact pressure) will be compared. The main differences between both the testing regimes are related to the real contact area between the counterparts and the possibility of entrapping lubricant between asperities. In the case of the microtests, due to the lower contact pressure, the presence of a lubricant film between asperities can be expected. Therefore, surface adsorption of the OFMs might play a more important role in the tribological response, wWhereas for the macrotests, due to the higher contact pressure and therefore the increased asperity contact, tribochemical reactions between the metal and the OFMs will dominate the tribological response.

Figure 6 shows the average coefficient of friction of the micro- and macrotests performed in this study. The graph has been split into different blocks to compare the different lubricants tested, i.e., WG and WGA alone, WG with C12 and monolaurin, and WGA with C12 and monolaurin. Interestingly, the friction in the macrotests is almost half of that in the microtests. At each contact condition, different mechanisms play a dominant role, i.e., surface adsorption vs tribochemistry. In the microtests, the role of surface roughness and adhesive forces is greater due to the lower contact pressure and the minor plastic deformation of the asperity contacts.^35,36^ In the macrotests, the effect of tribofilms, transfer layers, and third bodies is dominant since extensive plastic deformation and smearing of the mixed material takes place. Another interesting observation is the different friction trends in both contact situations. They are indeed the opposite for both additives when the amine is added to the lubricant.

When amine is not present in the base fluid, the best friction at both micro- and macrotests is found for WG containing 0.1 wt % C12. This is due to the good adsorption of C12 on the stainless steel surface (Figure 1). Indeed, C12 in WG forms a durable and rigid adsorbed layer, resulting in a low and steady friction throughout the test. Monolaurin in WG slightly adsorbs on the surface (Figure 1), and it is not effective enough, resulting in worse frictional performance. This is attributed to the lack of deprotonation for monolaurin in WG, which is clearly not reached at neutral pH (Table 2), thus hindering surface adsorption and resulting in higher friction.

The addition of 1 wt % amine to the water–glycol mixture (WGA) resulted in an increase in the microfriction, whereas there was no significant effect on the macrofriction. This increase in microfriction is in agreement with the adsorption results shown in Figure 5, where the amine molecules adsorb very efficiently to the surface of stainless steel. Therefore, the increase in microfriction can lead to the conclusion that the amine layer has a detrimental effect on microfriction. However, despite not having any significant effect in the macrofriction, it significantly reduced wear (Figures 3 and 4). Therefore, the contact mechanics and the tribochemical response of the system play roles in the tribological response.

The effect of the OFM concentration on friction was also studied in amine containing WG mixtures (WGA). Due to the presence of amine, microfriction increases for 0.1 and 0.2 wt % C12 concentration; however, it drastically decreases for 0.5 wt % C12. Looking at the adsorption results presented in Figure 5, the effect of competitive adsorption between the amine and C12 is obvious at the lowest concentrations, where the frequency graphs do not show any significant drop. In the case of 0.5 wt % C12, the concentration is well above the CMC, and this is reflected in the frequency drop in Figure 5. Therefore, the C12 micelles win the competition for the surface adsorption sites resulting in the lowest microfriction.

**Figure 7:**
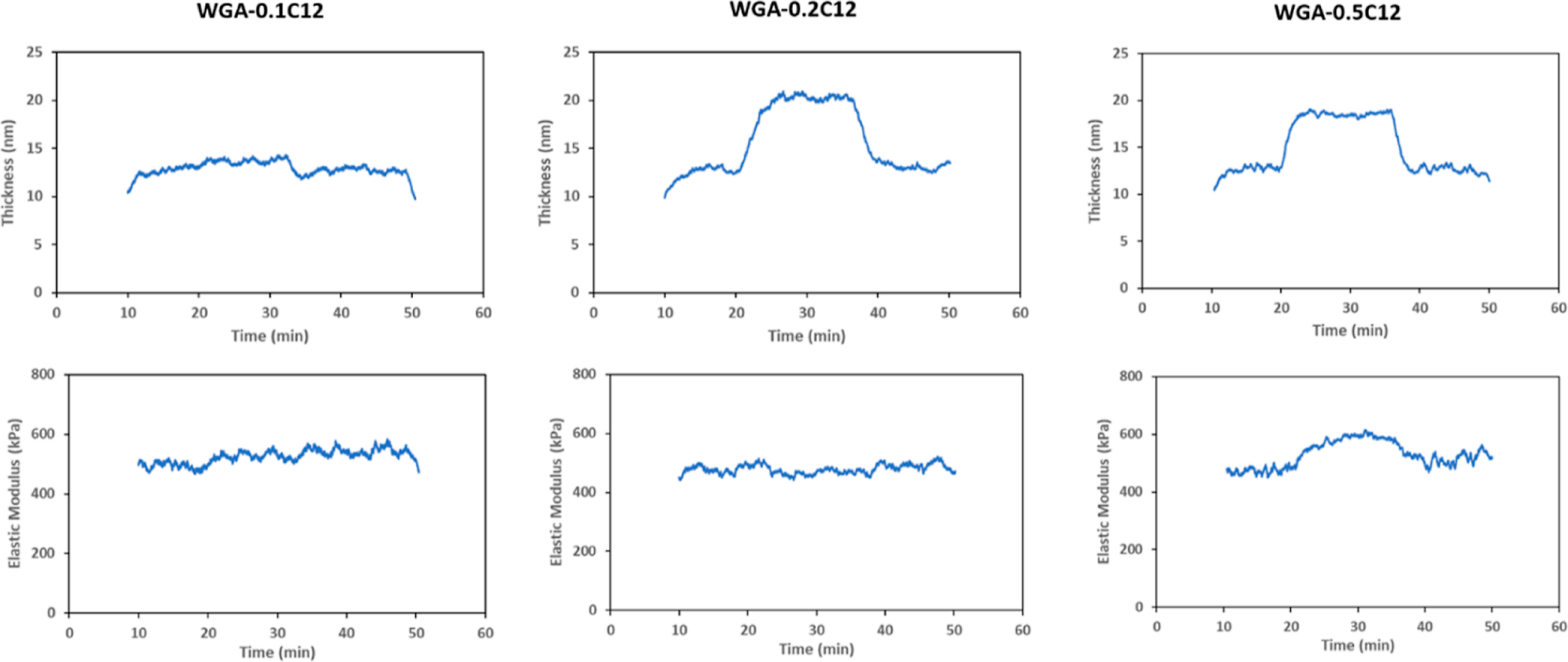
Results of the viscoelastic modeling for QCM measurements for different concentrations of C12 in the WGA.

Modeling the viscoelastic properties of the adsorbed layers was calculated based on the QCM experiments performed under no pressure (Figure 5). Data for the different concentrations of C12 in WGA (Figure 7) show interesting results. On one hand, the addition of 0.1 wt % of C12 to WGA does not have any effect on the viscoelastic properties and thickness as it can be identified by the small frequency drop in the QCM data (Figure 5). In the case of WGA-0.2C12, an increase of 20 nm in the thickness is found (Figure 7), but the viscoelastic properties remain the same, indicating that the amine wins the adsorption competition at this concentration. On the other hand, for 0.5 wt % C12 in WGA, a fast layer buildup is found, together with an increase in the elastic modulus to values around 600 kPa. Since 0.5 wt % C12 is above the CMC, the micelle formation is the reason for the change in the adsorption behavior. Moreover, the improvement of the mechanical behavior of the adsorbed layer can explain the lower microfriction for 0.5 wt % C12 compared to all other C12 concentrations (Figure 6).

**Figure 8:**
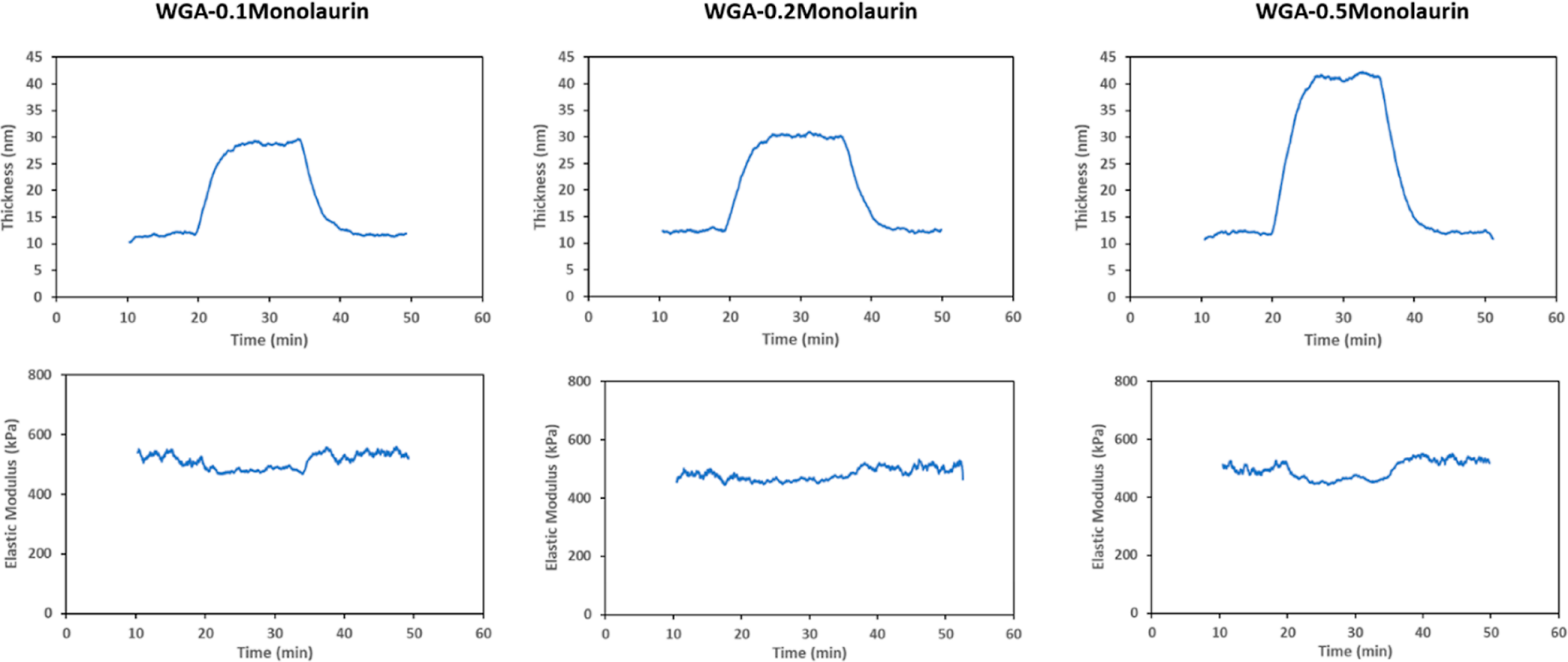
Results of the viscoelastic modeling for QCM measurements for different concentrations of monolaurin in the WGA.

In the case of monolaurin in WGA, no competition with the amine for the surface adsorption sites takes place, as can be seen from the large frequency drop for all concentrations in Figure 5. This can be explained by the fact that the pH of WGA containing monolaurin is well above the pH for the onset of deprotonation, which when combined with its very high dipole moment enhances surface adsorption (Tables 1 and 2). Figure 8 shows the results of the viscoelastic modeling for different concentrations of monolaurin in WGA. The increase in concentration results in an increase in the thickness of the adsorbed layer forming a multilayer arrangement on the surface, whereas the elastic modulus remains the same or slightly decreases (Figure 8). This agrees with the friction results found for the microtests where no significant changes in friction are observable for monolaurin at any of the three concentrations, and friction remains rather high, especially compared to C12 at 0.5 wt % (Figure 6). This indeed shows the importance of the elastic properties of the adsorbed layer on the frictional response, especially at the milder mechanical contact conditions of the microtests. For the macrotests, tribochemistry activated by the high contact pressure between the rubbing parts plays a more important role, and therefore, the trends in friction are different.

**Figure 9:**
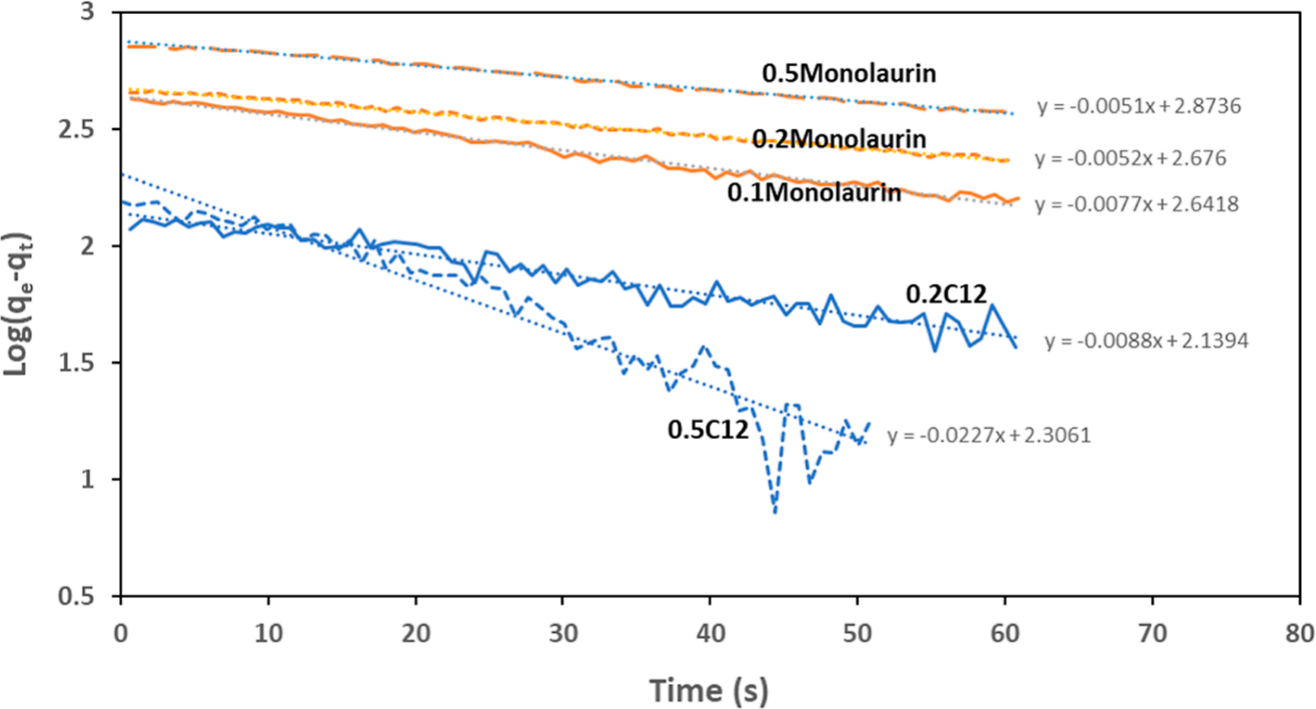
Time-dependent adsorption kinetics of different concentrations of C12 and monolaurin in WGA during the first 60 s of pumping the additivated WGA solutions–extracted from the graphs shown in Figure 6.

These different microfriction results between C12 and monolaurin can also be explained by studying the adsorption kinetics, which is another important parameter that explains the frictional behavior of the samples. The changes in the adsorbed mass versus time can be fit by a first-order two-dimensional adsorption kinetics equation as
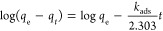
where *q*_*t*_ and *q*_e_ are the adsorbed mass at time t and in equilibrium (in ng/cm^2^), respectively, and *k*_ads_ is the adsorption constant (in s^–1^). Figure 9 shows log(*q*_e_ – *q*_*t*_) during the first 60 s of adsorption for 0.2 and 0.5 wt % C12, and 0.1, 0.2, and 0.5 wt % monolaurin in WGA. These data are extracted from the adsorption curves shown in Figure 5. The reason adsorption kinetics is not plotted for 0.1 wt % C12 is that adsorption competition between the amine and C12 takes place at this concentration, resulting in very small mass changes due to the balance established between adsorption of C12 and desorption of amine. Therefore, the adsorption kinetics might not be representative of C12 at this concentration.

The linear trendlines fitted to the adsorption kinetic graphs can be used to extract the adsorption constant for each lubricant. Comparing the adsorption constants, a significantly faster adsorption is found for 0.5 wt % C12 compared to all other lubricant formulations, which can be attributed to the micelle formation. The faster adsorption kinetics and the better viscoelastic properties at this concentration are responsible for its exceptional microfrictional response (Figure 6). For monolaurin, the adsorption kinetics remained very similar at all concentrations, the same as the viscoelastic properties; thus, no significant changes in microfrictional response were found.

For the macrotests, the effect of concentration was not as pronounced for C12 with friction values very low for all concentrations, being only slightly lower for 0.2 and 0.5 wt % C12 (Figure 6). Interestingly, the macrofriction values for monolaurin show high friction at 0.1 and 0.2 wt % despite the formation of a thicker adsorbed film (Figure 8) and very low friction at 0.5 wt %. For monolaurin, there is no micelle formation, there is no adsorption competition with amine, the viscoelastic properties of the layer are not good and do not significantly change with concentration, and slow adsorption kinetics are found as compared to C12. However, the thickness of the adsorbed layer significantly increases with concentration (Figure 8). This indicates that monolaurin forms a multilayer arrangement on the surface. Thus, it can be concluded that monolaurin must reach a certain thickness to have any effect on macrofriction. However, this alone will not explain the exceptional friction and wear performance of 0.5 wt % monolaurin in WGA. In the macrotests, additional effects take place, such as the high pressure and temperature in the contact, leading to tribochemical processes resulting in tribofilm formation. Indeed, this is evident comparing the wear results in Figures 2–4, especially for WGA containing 0.5 wt % monolaurin, which is the surfactant leading to the lowest wear rate. The effect of the tribofilms in the macrofriction and wear will be further investigated and discussed in the next section.

### Effect of the OFM Type on the Tribofilm Formation

**Figure 10:**
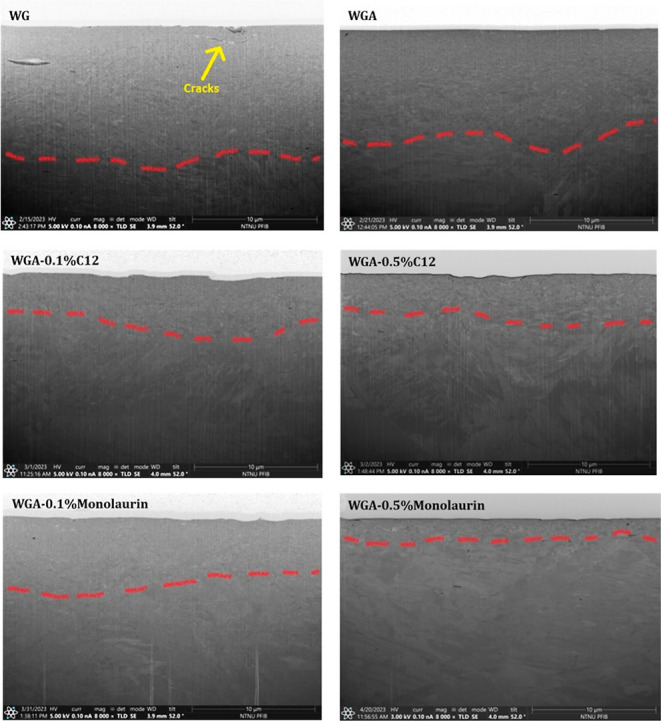
FIB prepared cross-section 8000× magnified images from the wear tracks of the samples tested in WG, WGA, WGA0.1C12, WGA0.5C12, WGA0.1Monolaurin, and WGA0.5Monolaurin. The red dashed lines denote the limit between the highly recrystallized regions and bulk of the material. The yellow arrows indicate where cracks have been formed.

Figure 10 shows the FIB cross-section images taken from the wear tracks of the macrotests performed in WG, WGA, WGA0.1C12, WGA0.5C12, WGA0.1Monolaurin, and WGA0.5Monolaurin.

The cross-section image of the sample tested in WG shows a high level of recrystallization and many cracks on the surface and subsurface regions. The high recrystallization level is in good agreement with the high coefficient of friction during the test (∼0.26), and the formation and propagation of the cracks agree with the high wear rate found for this sample (Figure 3). The cross-section of the sample tested in WGA shows a high recrystallization level (similar to WG), which agrees well with its high coefficient of friction, but the top surface is quite smooth and crack-free, which can be an indicator of this sample’s lower wear rate compared to WG (Figure 4). The cross-section of the sample tested in WGA0.1C12 shows a very small, recrystallized region compared to the two previous samples, which agrees well with its low coefficient of friction (∼0.14). In the case of WGA0.5C12, an even smaller recrystallized area is found, which is in line with the even lower friction (∼0.11) for this sample. The recrystallization level increases in the case of WGA0.1Monolaurin compared to the lubricants formulated with C12, and this is in good agreement with the slightly higher friction of this sample compared to C12 (∼0.22). WGA0.5Monolaurin shows the smallest recrystallized area and the smoothest surface finish in the cross-section, which is in good agreement with its lowest macrofriction and wear of all lubricants tested.

**Figure 11:**
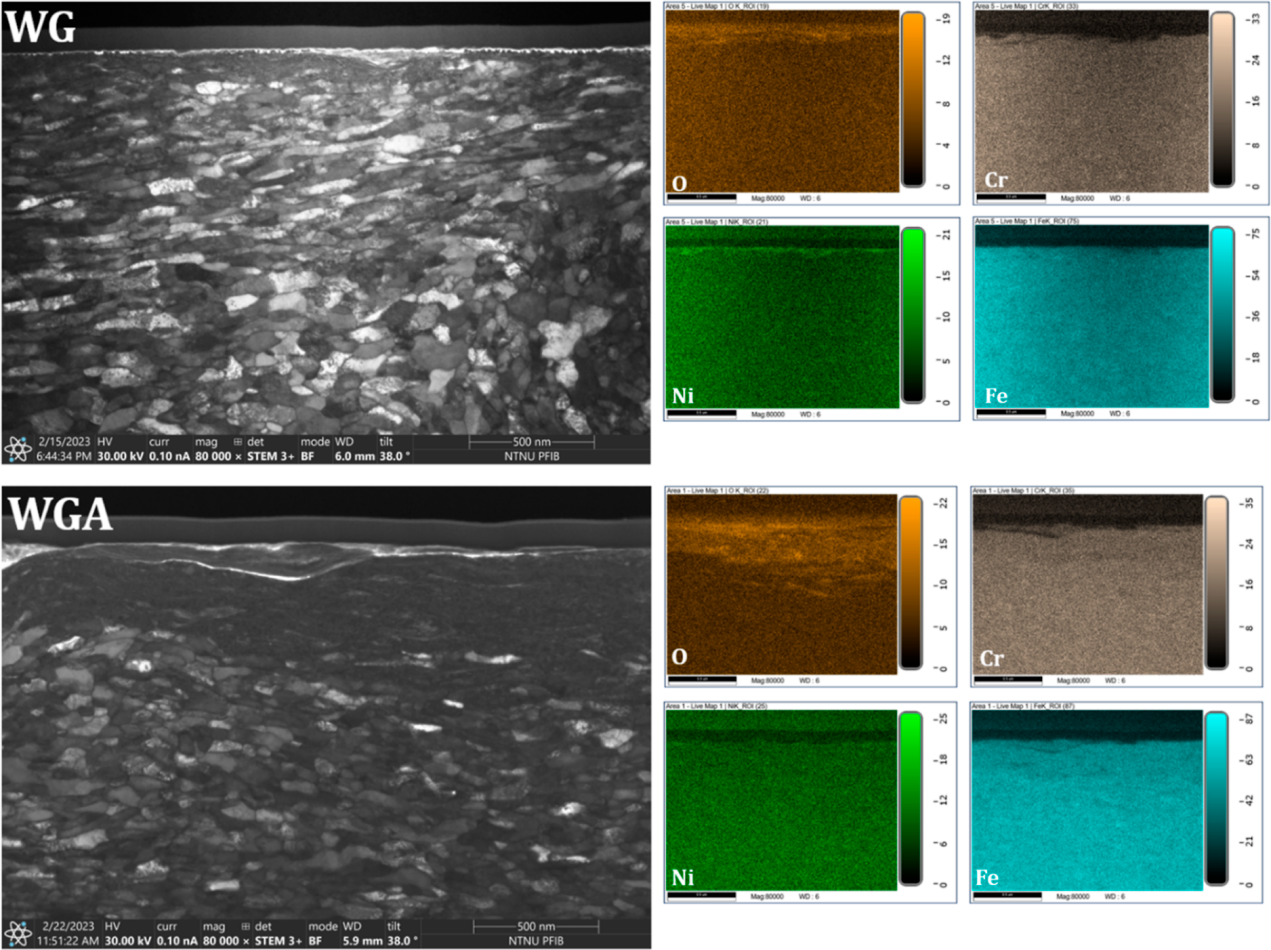
STEM 80 000× magnified images from the wear tracks of the samples tested in WG and WGA; EDS elemental maps.

For further investigation of the tribofilm formation and its effect on friction and wear, FIB-STEM-EDS characterization was performed. Very thin (<60 nm) TEM lamellae were prepared from the wear track of the samples tested in WG, WGA, WGA0.1C12, WGA0.5C12, WGA0.1Monolaurin, and WGA0.5Monolaurin. Figure 11 shows the STEM image of the subsurface area and the EDS elemental maps of O, Cr, Ni, and Fe for the wear track of the samples tested in WG and WGA alone.

The STEM image from the wear track of WG does not show any sign of tribofilm formation, and only a thin ultrafine-grained (with a thickness of about 100 nm) layer with some oxygen in the EDS map is detectable. This layer consists of highly deformed nanocrystalline grains and some oxide smeared in the structure. In the case of the sample tested in WGA, a discontinuous oxide film is embedded in a thick, ultrafine grained structure, most likely formed as a result of a tribochemical reaction of the amine with the metal alloy. The thickness of this layer is around 600 nm. Indeed, WGA resulted in much lower wear than WG (Figures 3 and 4), which by the STEM results, it can be attributed to the formation of this thick ultrafine-grained layer mixed with oxides.

**Figure 12:**
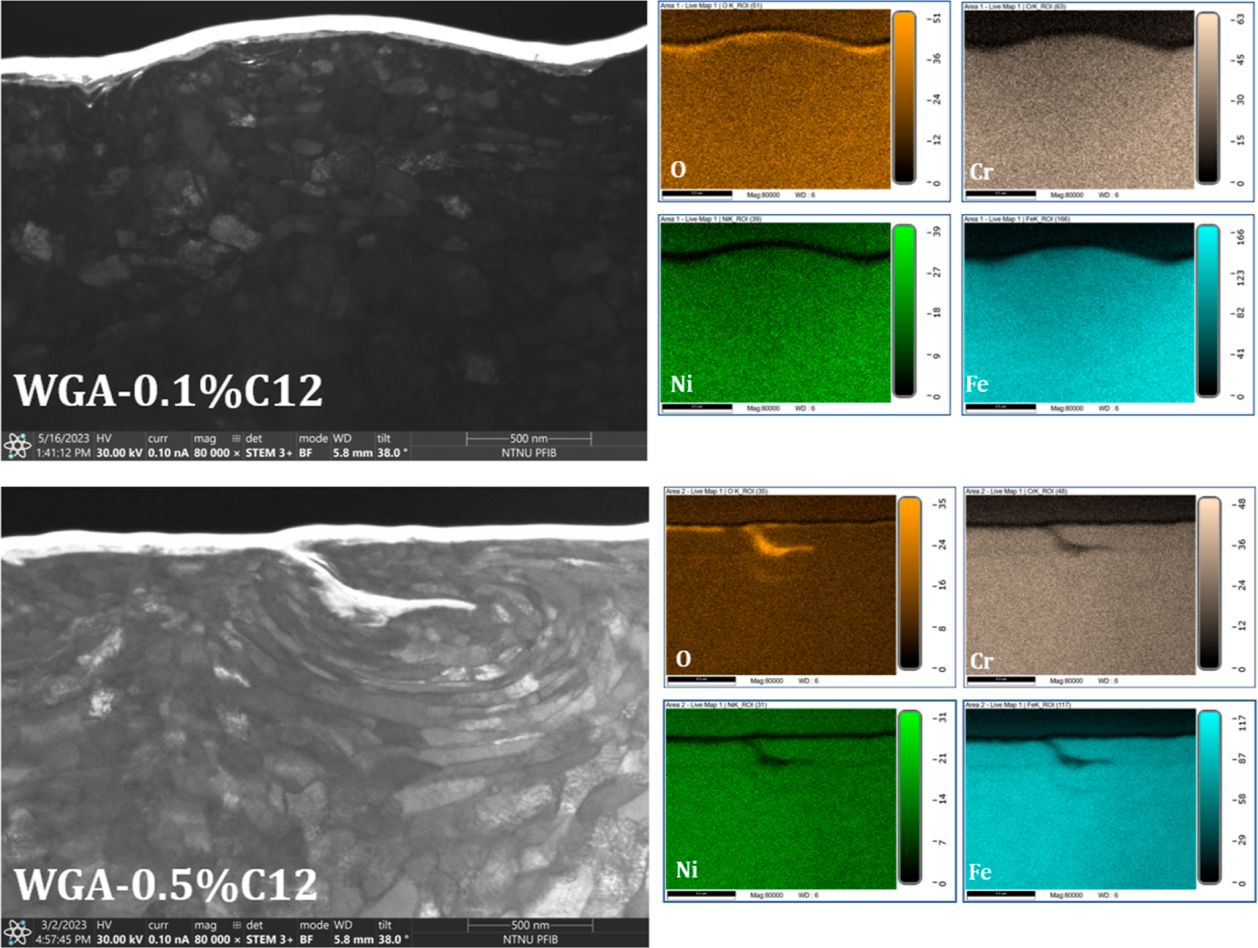
STEM 80 000× magnified images from the wear tracks of the samples tested in WGA0.1C12 and WGA0.5C12; EDS elemental maps.

When the surfactants are added to WGA, tribofilm formation depends on the type and concentration of the additive. Figure 12 shows the STEM image of the subsurface area and EDS elemental maps for the wear track of the samples tested in WGA0.1C12 and WGA0.5C12.

The STEM image of the sample tested in WGA0.1C12 shows a very thin and continuous oxide film on the top surface. On the other hand, the STEM image of the sample tested in WGA0.5C12 illustrates an oxide film which is partially smeared on the subsurface region. Moreover, the oxygen EDS map of this sample shows a discontinuous film. The better surface coverage of the oxide film formed on the sample tested in WGA0.1C12 can be the reason for its lower β value compared to that of WGA0.5C12 (Figure 4). As shown in Figure 4, the macrofriction is drastically affected by adding different concentrations of C12 to the base WGA, but it does not significantly reduce wear (resulting in even higher wear in the case of WGA0.1C12). This indicates that the oxide films formed by C12 do not have any antiwear functionality.

**Figure 13:**
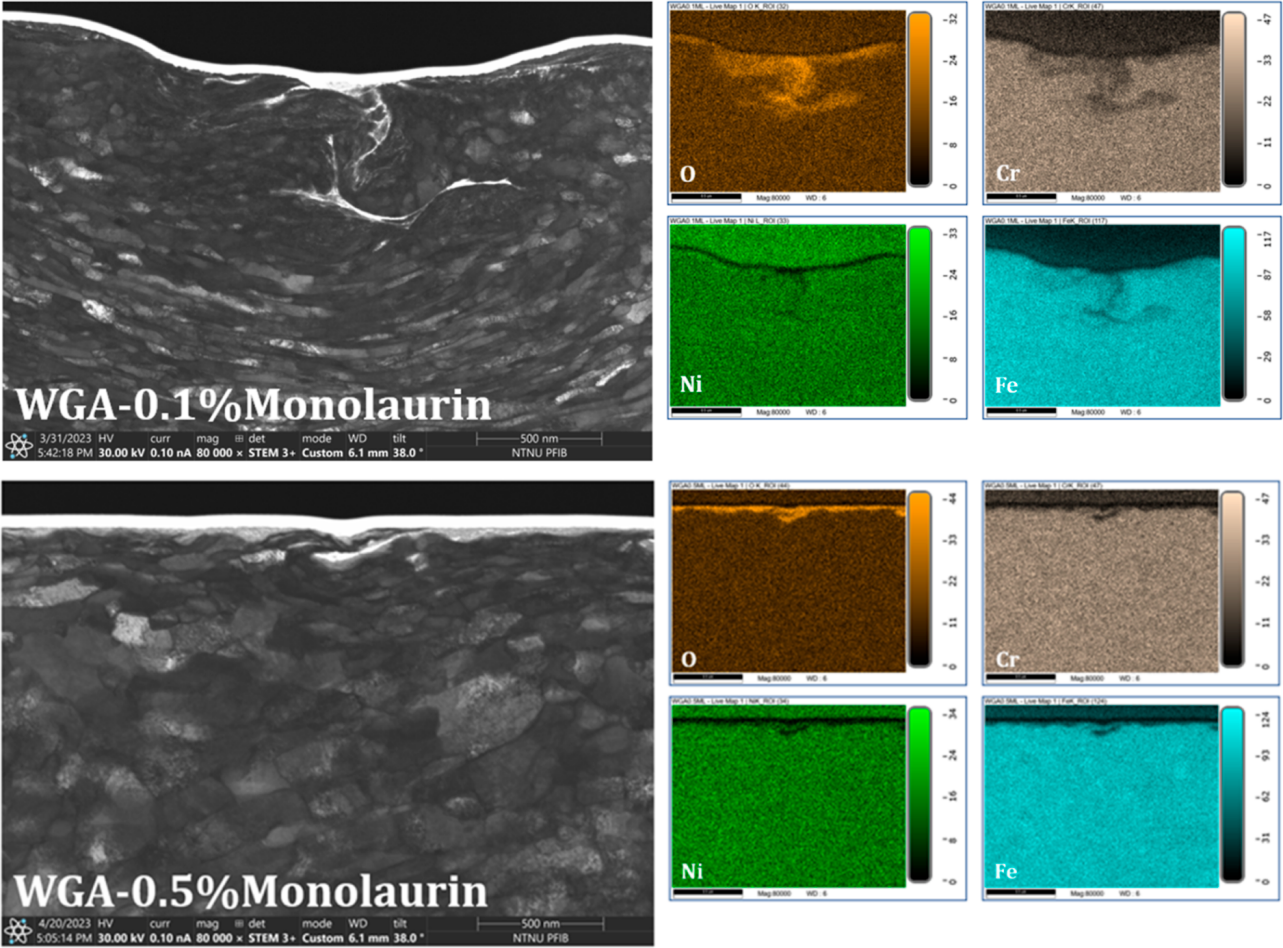
STEM 80 000× magnified images from the wear tracks of the samples tested in WGA0.1Monolaurrin and WGA0.5Monolaurin; EDS elemental maps.

The thicker but poorer viscoelastic properties of monolaurin result in higher macrofriction, except for 0.5 wt % concentration, which also results in the best wear performance. Figure 13 shows the STEM image and EDS elemental maps for the wear track of the samples tested in WGA0.1Monolaurin and WGA0.5Monolaurin. The STEM image of the sample tested in WGA0.1Monolaurin shows a rough surface with discontinuous oxide films smeared on the subsurface region. Indeed, this sample has resulted in higher wear than that of WGA alone. On the other hand, the STEM image of the sample tested in WGA0.5Monolaurin shows a homogeneous oxide film covering the surface. Moreover, the top surface is very smooth, and the grain size of the subsurface region is bigger than that of the other samples, which is in good agreement with the very low macrofriction response of this sample. This oxide-rich tribofilm is thin but protective, providing good antiwear functionality to this surfactant.

## Conclusions

Three different surfactants (lauric acid, methyl laurate, and monolaurin) with the same tail structure and different heads were studied in a water/glycol base lubricant. The adsorption investigations showed that methyl laurate does not effectively adsorb on the surface of the stainless steel-coated QCM sensors. Therefore, detailed micro- and macrotribological tests were performed only with C12 and monolaurin. 1 wt % of *N*,*N*-dimethylethanolamine was added to the base fluid, allowing the surfactants of higher concentrations to dissolve. The surface and subsurface of the wear tracks obtained in macrotests were studied using SEM, FIB, STEM, and EDS. The main conclusions derived from this work can be summarized as follows:

- The adsorption investigations by QCM showed that C12 competes with amine for the surface adsorption sites on stainless steel. At lower concentrations (0.1 wt %), amine is the winner of the competition, but for concentrations higher than the critical micelle concentration (0.5 wt %), a big frequency drop was observed. In the case of monolaurin, the adsorption graphs did not show any competitive adsorption. The frequency evolution graphs showed almost the same adsorption at the concentrations of 0.1 and 0.2 wt % and higher adsorption at 0.5 wt %, which is attributed to the full deprotonation and the very high dipole moment of this molecule in the water–glycol-amine base lubricant.
- Modeling the thickness and viscoelastic properties of the adsorbed layers of C12 and monolaurin revealed that monolaurin more efficiently adsorbs on the stainless steel forming multilayers. However, this was not translated in any improvement of the microfriction. The best viscoelastic properties were found for C12 at 0.5 wt % concentration due to the micelle formation, thus resulting in the lowest microfriction. This was further confirmed by the adsorption kinetics where 0.5 wt % C12 resulted in the fastest adsorption kinetics of all lubricants. Therefore, for tribosystems running in mild boundary lubricating conditions, the viscoelastic properties of the adsorbed layers, rather than the thickness of the layer, play the most important role in the frictional response of the system.
- In the case of a tribosystem running in harsh contact boundary lubricating conditions, the most important role is the availability of the additive in the contact (thickness and mass of the adsorbed layer) to efficiently interact with the metal surface to form an effective tribofilm that can substantially reduce both friction and wear. This is indeed confirmed for the highest concentration (0.5 wt %) of monolaurin, which resulted in the lowest friction and wear among all the lubricants tested.
